# [Retracted] MicroRNA‑25 protects nucleus pulposus cells against apoptosis via targeting SUMO2 in intervertebral disc degeneration

**DOI:** 10.3892/mmr.2024.13212

**Published:** 2024-04-02

**Authors:** Changbin Lei, Jian Li, Guang Tang, Jiong Wang

Mol Med Rep 24: 724, 2021; DOI: 10.3892/mmr.2021.12363

Following the publication of this paper, the authors requested that the paper be retracted, specifically on account of deficiencies that were identified both in the documentation of patient records and in written consent pertaining to the data presented in Fig. 1.

After having considered the authors’ request, the Editor of *Molecular Medicine Reports* has agreed that this paper should be retracted from the Journal. All the authors are in agreement with the decision to retract this paper. The Editor apologizes to the readership for any inconvenience caused.

